# The Zebrafish *moonshine* Gene Encodes Transcriptional Intermediary Factor 1γ, an Essential Regulator of Hematopoiesis

**DOI:** 10.1371/journal.pbio.0020237

**Published:** 2004-08-17

**Authors:** David G Ransom, Nathan Bahary, Knut Niss, David Traver, Caroline Burns, Nikolaus S Trede, Noelle Paffett-Lugassy, Walter J Saganic, C. Anthoney Lim, Candace Hersey, Yi Zhou, Bruce A Barut, Shuo Lin, Paul D Kingsley, James Palis, Stuart H Orkin, Leonard I Zon

**Affiliations:** **1**Howard Hughes Medical Institute, Chevy ChaseMaryland, United States of America; **2**Division of Hematology/Oncology, Children's Hospital and Harvard Medical SchoolBoston, Massachusetts, United States of America; **3**Department of Molecular, Cell and Developmental BiologyUniversity of California, Los Angeles, California, United States of America; **4**Department of Pediatrics and Center for Human Genetics and Molecular Pediatric Disease, University of Rochester Medical CenterRochester, New YorkUnited States of America

## Abstract

Hematopoiesis is precisely orchestrated by lineage-specific DNA-binding proteins that regulate transcription in concert with coactivators and corepressors. Mutations in the zebrafish *moonshine (mon)* gene specifically disrupt both embryonic and adult hematopoiesis, resulting in severe red blood cell aplasia. We report that *mon* encodes the zebrafish ortholog of mammalian transcriptional intermediary factor 1γ (TIF1γ) (or TRIM33), a member of the TIF1 family of coactivators and corepressors. During development, hematopoietic progenitor cells in *mon* mutants fail to express normal levels of hematopoietic transcription factors, including *gata1,* and undergo apoptosis. Three different *mon* mutant alleles each encode premature stop codons, and enforced expression of wild-type *tif1γ* mRNA rescues embryonic hematopoiesis in homozygous *mon* mutants. Surprisingly, a high level of zygotic *tif1γ* mRNA expression delineates ventral mesoderm during hematopoietic stem cell and progenitor formation prior to *gata1* expression. Transplantation studies reveal that *tif1γ* functions in a cell-autonomous manner during the differentiation of erythroid precursors. Studies in murine erythroid cell lines demonstrate that Tif1γ protein is localized within novel nuclear foci, and expression decreases during erythroid cell maturation. Our results establish a major role for this transcriptional intermediary factor in the differentiation of hematopoietic cells in vertebrates.

## Abstract 2

A new gene acting early in red blood cell development is discovered by genetic analysis in zebrafish. The gene encodes a member of a well-known family of transcription factors

## Introduction

Hematopoiesis involves the coordinated processes of cell proliferation and differentiation of a relatively small number of progenitor cells into billions of circulating red and white blood cells (Thisse and Zon 2002). Hematopoiesis in vertebrates, from zebrafish to humans, is an evolutionarily conserved program that produces two waves of stem or progenitor cells that differ both in their embryonic origins and in the lineages of differentiated blood cells produced (Palis and Yoder 2001; Orkin and Zon 2002; Galloway and Zon 2003). The first, or primitive, wave of hematopoiesis originates from ventral mesoderm and gives rise to progenitor cells that differentiate in embryonic blood islands. The primitive wave of hematopoiesis produces a burst of embryonic erythrocytes and macrophages. The second, or definitive, wave of hematopoiesis arises from self-renewing stem cells that develop primarily in the intraembryonic aorta–gonad–mesonephros region. These definitive hematopoietic stem cells seed the later developing marrow spaces, to produce all lineages of adult blood cells, including definitive erythrocytes, myeloid cells, and lymphocytes.

We have undertaken a genetic approach to characterize genes that control hematopoiesis using the zebrafish as a model system (Thisse and Zon 2002). As part of a large-scale forward genetic screen, we previously identified bloodless zebrafish mutants that failed to express the erythroid transcription factor *gata1* normally in embryonic hematopoietic precursors (Ransom et al. 1996). We named one of these zebrafish genes *moonshine (mon),* and another group named a noncomplementing allele *vampire* (Weinstein et al. 1996).

Here, we have determined that mutations in the *mon* gene cause a disruption in both primitive embryonic and definitive adult hematopoiesis, resulting in a severe loss of erythroid cells. Erythroid progenitor cells in *mon* mutants are initially present, but fail to express normal levels of hematopoietic transcription factors and undergo apoptosis.

Positional cloning identifies the *mon* gene as the zebrafish ortholog of mammalian *transcriptional intermediary factor 1γ (TIF1γ),* a member of the TIF1 family of transcriptional coactivators and corepressors (Le Douarin et al. 1995; Friedman et al. 1996; Kim et al. 1996; Venturini et al. 1999; Peng et al. 2002). The three members of the vertebrate TIF1 family (α, β, and γ) are large nuclear proteins that each contain an N-terminal RBCC or TRIM domain (Reymond et al. 2001) composed of a RING finger, two B-boxes, and a coiled-coil domain. TIF1 family members also contain a C-terminal plant homeodomain finger and bromodomain that are characteristic of chromatin remodeling factors. TIF1α has been shown to associate with a variety of ligand-bound nuclear hormone receptors (Le Douarin et al. 1995) and function as a coactivator for retinoic acid receptors (Zhong et al.1999). TIF1β has been shown to act as a corepressor for the large family of Krüppel-associated box (KRAB) domain zinc-finger transcription factors (Friedman et al. 1996; Abrink et al. 2001). In contrast, TIF1γ does not associate directly with either nuclear receptors or KRAB domains that bind to the other TIF1 family members (Venturini et al. 1999; Abrink et al. 2001). Biochemical studies also demonstrate that TIF1γ forms both homo-oligomers and hetero-oligomers with TIF1α but not with TIF1β (Peng et al. 2002). The murine *Tif1α* and *Tif1γ* genes have not yet been subjected to gene targeting experiments, whereas analysis of mouse mutants demonstrates that *Tif1β* is required for postimplantation embryogenesis and mesoderm induction in particular (Cammas et al. 2000). Taken together, these studies suggest that a major function of TIF1 family members is to link DNA-binding proteins with other coactivators or corepressors during development.

Our studies establish that *tif1γ* functions as an essential regulator of embryonic and adult hematopoiesis in vertebrates. Cell transplantation studies demonstrate that *tif1γ* acts in a cell-autonomous manner during embryonic hematopoiesis. The *tif1γ* gene is expressed specifically in ventral mesoderm and hematopoietic progenitors, then downregulated as erythroid maturation occurs. Tif1γ protein localizes to a novel class of nuclear bodies in both primary mouse embryo fibroblasts and erythroleukemia cell lines. Taken together, our studies demonstrate that Tif1γ is required for normal erythroid cell development and survival.

## Results

### The Zebrafish *mon* Gene Is Essential for Both Primitive and Definitive Erythropoiesis

**Figure 1:**
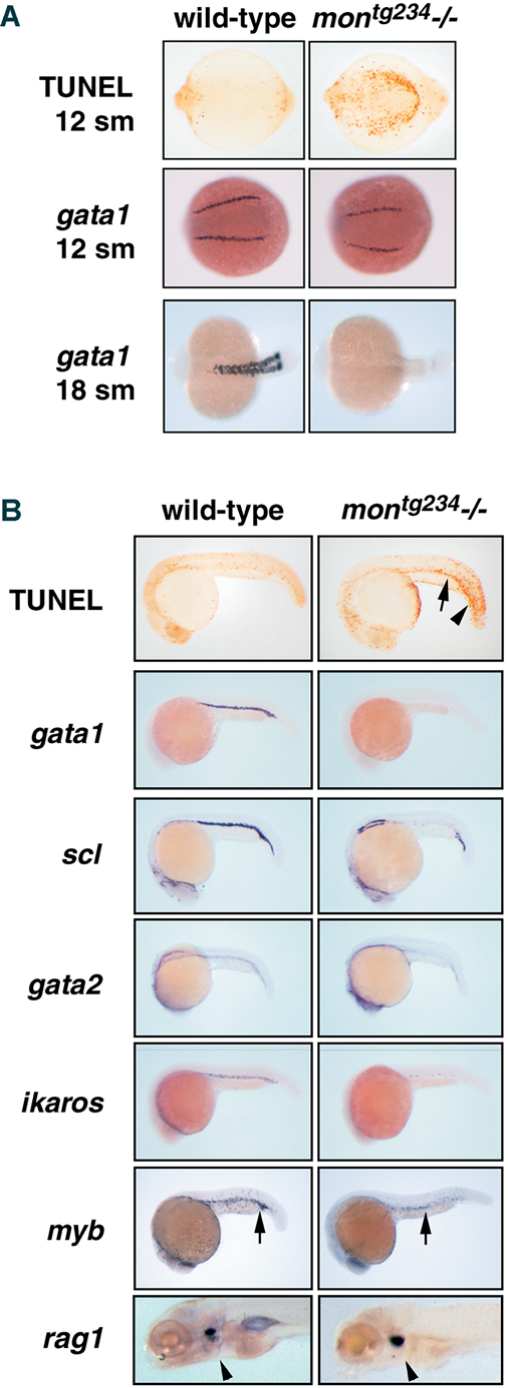
Zebrafish *mon* Mutants Have Severe Defects in Primitive Hematopoiesis (A) Whole-mount TUNEL assays reveal that ventral-posterior mesodermal cells undergo apoptosis in homozygous *mon^tg234^* mutant embryos. Whole-mount in situ hybridization of *gata1* detected at the 12- and 18-somite stage in genotyped embryos. Posterior views, anterior to the left. (B) Extensive apoptosis is visible in the trunk and tail (arrowhead) and also in hematopoietic cells of the embryonic blood island at 22 h of development (arrow). Whole-mount in situ hybridization at 22 hpf including *scl, gata2, gata1, ikaros,* and *myb* in *mon^tg234^* mutants. Expression of *myb* is greatly reduced in the blood islands because of a loss of erythroid cells, but embryonic macrophages are still present (arrows). The expression of *rag1* in thymic T-cells appears normal in *mon* mutants at 5 d postfertilization (arrow heads). Lateral views of 22 hpf and 5-d-old embryos.

In order to determine when the *mon* gene is required in development, we first examined hematopoietic gene expression and apoptosis in zebrafish homozygous *mon* mutant embryos. During embryogenesis, homozygous zebrafish *mon* mutants have no red blood cells (RBCs) visible in circulation (Ransom et al. 1996; Weinstein et al. 1996). The *mon* mutants initiate expression of *gata1* in hematopoietic cells around the five-somite stage, similar to wild-type embryos (data not shown); however, based on TUNEL staining, the differentiating erythroid cells undergo programmed cell death from the 12-somite stage to 22 h postfertilization (hpf) (Figure 1A and 1B, arrows). At 12 somites, *gata1* expression is only slightly reduced. By 18–22 hpf, hematopoietic-specific markers such as *gata1, scl, gata2,* and *ikaros* are not detected in the embryonic blood island (Figure 1A and 1B; unpublished data). The hematopoietic cells are thus correctly specified early during the development of *mon* mutant embryos, but these precursors undergo cell death. Based on expression of c-*myb* and *rag1* (Figure 1B, arrows), *mon* mutants have normal myeloid and lymphoid development, respectively. In addition to the deficit of RBCs in *mon* mutants, there is a prominent loss of fin-fold and tail mesenchyme (Ransom et al. 1996). TUNEL staining of *mon* mutants demonstrates extensive apoptosis of mesenchymal cells in the trunk and tail bud regions (Figure 1A and 1B, arrows). The *mon* gene is thus required for normal development and survival of both committed erythroid progenitor cells and posterior mesenchymal cells.

**Figure 2:**
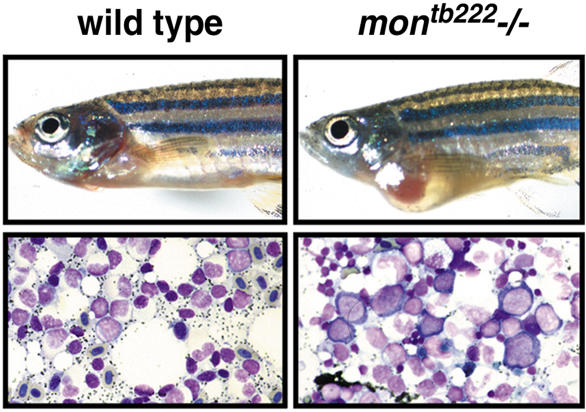
Zebrafish *mon* Mutants Also Have Severe Defects in Definitive Hematopoiesis Adult phenotype of wild-type and *mon* mutants. A rare surviving *mon^tb222^* homozygous adult shows significant cardiomegaly in comparison to a wild-type age-matched control. Wright–Giemsa stained marrow of wild-type adult in comparison to a homozygous mutant. Note the dramatic reduction of terminally differentiated erythroid cells and the presence of abnormally large megaloblastic proerythroblasts in the *mon^tb222^* mutant marrow.

We next examined definitive hematopoiesis in rare surviving homozygous adult zebrafish *mon* mutants. Mutations in *mon* are generally lethal by 10 to 14 d of development (Ransom et al. 1996), although rare *mon* homozygous mutants (approximately 1 in 500 bloodless embryos) of all tested alleles survive to adulthood. Adult *mon* mutants show cardiac hypertrophy, presumably due to the severe anemia leading to a high output state (Figure 2). In wild-type zebrafish, the adult site of hematopoiesis is the kidney (Al Adhami and Kunz 1977), which contains erythroid, lymphoid, and myeloid populations at various stages of differentiation (Bennett et al. 2001). In *mon* homozygous mutants, there is a severe block in maturation at the proerythroblast stage (Figure 2), whereas the differentiation of myeloid cells is normal (unpublished data). This demonstrates that the *mon* gene product acts during both primitive and definitive erythropoiesis.

### Positional Cloning Identifies *mon* as the Zebrafish Ortholog of Mammalian TIF1γ

**Figure 3:**
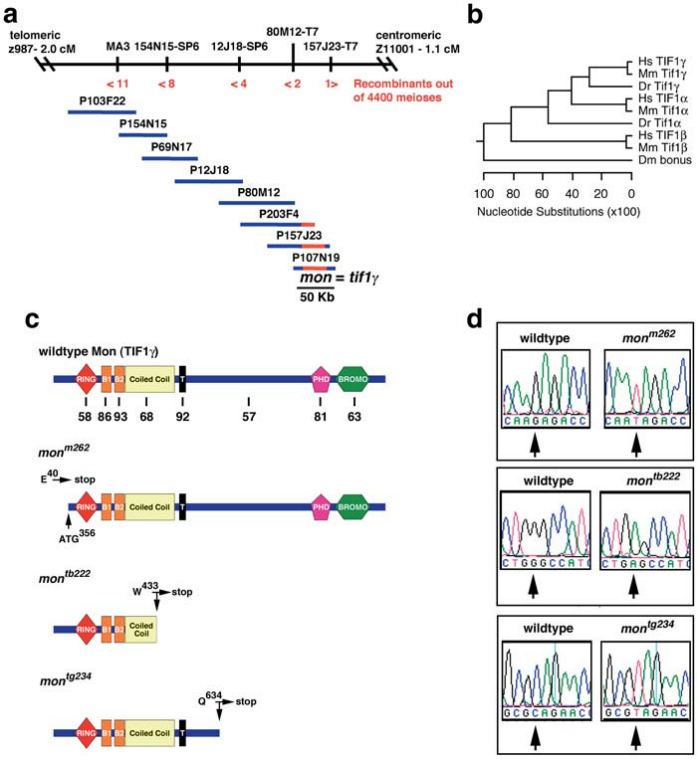
Positional Cloning Identifies the *mon* Gene as Zebrafish *tif1γ* (A) Physical map of the *mon* locus on zebrafish Chromosome 8. Microsatellite markers z987 and z11001 were used to initially identify recombinants in a panel of 2,200 diploid *mon^tg234^* homozygous mutants. The AFLP marker MA3 was used to initiate a chromosomal walk in PAC libraries. The critical PACS that were isolated to encompass the *mon* locus are indicated by numbers above bar. The PAC 107N19 defines the critical interval for the *mon* gene. This PAC was used as a probe to screen cDNA libraries and to identify zebrafish *tif1γ* cDNAs. Numbers below the bar indicate the number of recombinants identified by SSCP analysis. (B) Clustal-W–generated phylogentic tree of zebrafish (Danio rerio [Dr]) Tif1γ and Tif1α peptide sequences in comparison to TIF1 family members: human (Hs) TIF1α, TIF1β, and TIF1γ; mouse (Mm) Tif1α, Tif1β, and Tif1γ;; and fly (Dm) bonus. (C) Diagrams illustrating the structure of the Tif1γ-predicted peptide and the three identified point mutants. RING finger (RING), B-boxes (B1 and B2), plant homeodomain finger (PHD) and bromodomain (BROMO). Numbers below the first diagram indicate the percent identity shared between each of these domains in zebrafish and human TIF1γ. The predicted truncated proteins are indicated. (D) DNA sequence chromatograms showing the three ENU-induced point mutants in comparison to wild-type control sequences

We identified the *mon* gene by positional cloning using a panel of 2,200 diploid mutants collected from Tübingen background (TU)/WIK strain hybrid parents carrying the *mon^tg234^* allele. The *mon* mutant gene was positioned on Chromosome 8 between microsatellite markers z987 and z11001 (Figure 3A) (Knapik et al. 1998). For positional cloning purposes, over 12,000 polymorphic markers were screened using amplified fragment length polymorphism (AFLP) (Ransom and Zon 1999), and 36 markers within the interval were isolated. One of these, MA3, was found to be 0.3 cM from the gene (Figure 3A) and was utilized as the starting point of a chromosomal walk. A critical P1 bacterial artificial chromosome clone (PAC), 107N19, was obtained that spanned the genetic interval. Two simple sequence conformation polymorphism (SSCP) markers found on this PAC clone flank the critical genetic interval. The marker 80M12-T7 maps two recombinants out of 4,400 meioses telomeric of the mutation, and the marker 157J23-T7 maps one recombinant centromic of the mutation (Figure 3A). The end sequences and SSCP markers of PAC 107N19 are found in the zebrafish genomic sequence contig ctg23107 (http://www.ensembl.org/Danio_rerio/) containing a predicted zebrafish TIF1 family gene. This PAC was hybridized to a kidney cDNA library, resulting in the isolation of four clones that represented the same gene.

The *mon* gene encodes a member of the TIF1 family of transcriptional cofactors (Figure 3B and 3C). The coding sequence of *mon* is most similar to human TIFγ (Le Douarin et al. 1995; Friedman et al. 1996; Venturini et al. 1999), and the locations of exon boundaries are conserved between the zebrafish and human genes (unpublished data). The *mon* locus on zebrafish Chromosome 8 is also predicted to be syntenic to the region of human Chromosome 1p that contains the *TIF1γ* gene based on the conserved locations of 12 other orthologous gene pairs, including *NRAS,* mapped to these regions in human and zebrafish (Barbazuk et al. 2000). Therefore, based on sequence similarity and chromosomal location, the zebrafish *mon* gene is the likely ortholog of the human *TIF1γ* gene.

We have identified ethyl-nitrosourea (ENU)-induced point mutations in three alleles of *mon* (Figure 3C and 3D), each of which generates a premature stop codon. The *mon^tb222b^* and *mon^tg234^* alleles have a severe phenotype with no circulating blood cells. In contrast, the *mon^m262^* allele has 10–100 circulating blood cells by 48 hpf, in comparison to the approximately 3,000 RBCs in the circulation of wild-type or heterozygous embryos at the same time point. The *mon^m262^* allele was found to encode a premature stop codon at position E40, which would encode a putative protein of only 40 amino acids. Although this mutation would be expected to lead to a complete loss of *mon* gene product, another methionine is found downstream at amino acid position 104. In vitro translation experiments in reticulocyte lysates demonstrate reinitiation of translation from this methionine (unpublished data). Therefore, the hypomorphic larval phenotype of the *mon^m262^* allele is likely due to partial loss of *mon* function or expression. The presence of mutations in each of the *mon* alleles indicates that defective Tif1γ function is the cause of the *mon* phenotype.

**Figure 4:**
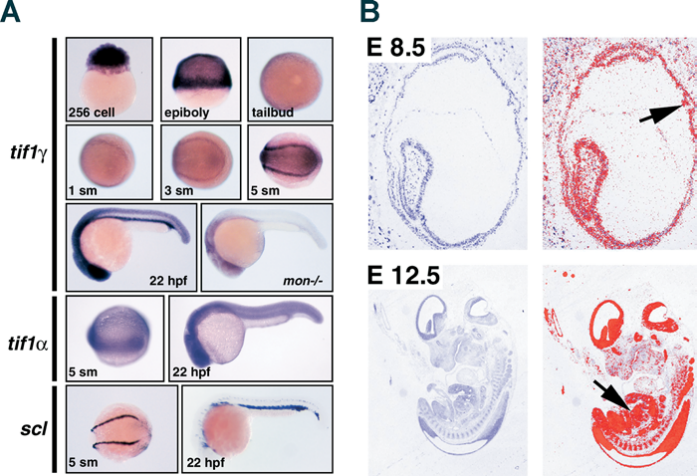
The *mon/tif1γ* Gene Is Highly Expressed in Hematopoietic Mesoderm (A) In situ hybridization of zebrafish embryos demonstrating the embryonic expression of *tif1γ. tif1γ* is initially expressed as a maternal mRNA. Increased expression of *tif1γ* in ventral-lateral mesoderm begins between the one- to three-somite stages and increases through early development. By five somites, *tif1γ* is strongly expressed in lateral stripes of mesoderm that also express *scl.* At 22 hpf *tif1γ* is expressed broadly in the brain, spinal cord, trunk, and tail mesenchyme, but is at much higher levels in hematopoietic cells of the blood island. Zebrafish *tif1α* is also broadly expressed but relatively more uniform in most tissues, in comparison to *tif1γ. Tif1α* is weakly expressed at early somite stages in hematopoietic mesoderm and uniformly expressed at 22 hpf, including expression in the blood islands. Expression of *scl* at five somites and 22 hpf highlights the embryonic blood island in comparison to *tif1γ* expression. (B) In situ hybridization of mouse embryos detects broad expression of *Tif1γ* at embryonic day 8.5 including the yolk sac blood islands (arrow). AT embryonic day 12.5, there is high level expression in the fetal liver (arrow) and broad expression in the embryonic brain, spinal chord, gut, and muscle.

In order to determine whether *tif1*γ is expressed in hematopoietic mesoderm, we next examined zebrafish embryos by whole-mount in situ hybridization (Figure 4A). *tif1γ* mRNA is expressed maternally and is found throughout the embryo during blastula stages. During gastrulation and epiboly stages, zygotic expression of *mon* is highest in the mesendoderm of the germ ring. At tail bud and early somite stages a high level of *tif1γ* expression delineates a horseshoe-shaped population of ventral/lateral mesoderm that will give rise to blood and also expresses *stem cell leukemia*hematopoietic transcription factor *(scl)* (Liao et al. 1997). This group of cells continues to express *tif1γ* and *scl* while it converges and forms the embryonic blood island (Detrich et al. 1995). The *tif1γ* gene is also highly expressed in the central nervous system as well as the mesenchyme of the trunk and tail. Homozygous *mon^tg234^* mutants have a greatly reduced amount of *tif1γ* mRNA in all tissues consistent with nonsense-mediated message decay. Thus, zebrafish *tif1γ* is specifically expressed in ventral mesoderm and putative hemangioblasts prior to and during the embryonic stages when hematopoietic progenitors are undergoing apoptosis in *mon* mutants. We also compared the expression of zebrafish *mon* to mouse *Tif1γ* (Figure 4A and 4B). Mouse *Tif1*γ is highly expressed in erythroid blood islands of the yolk sac, and it is subsequently expressed in the fetal liver at a high level, and in other tissues, including the central nervous system. Taken together these results strongly suggest that zebrafish *mon* and mouse *Tif1γ* are orthologs that function during hematopoiesis.

Given that mammalian TIF1γ has been shown to form hetero-oligomers with Tif1α (Peng et al. 2002), we searched for additional TIF1 family members in zebrafish to compare with *tif1γ.* Using zebrafish expressed sequence tag (EST) sequences, we designed primers to RT-PCR amplify a TIF1-related cDNA from embryonic 10-hpf and 24-hpf RNA. This cDNA encodes a predicted zebrafish ortholog of human TIF1α based on predicted amino acid sequences (see Figure 3B). In addition, zebrafish *tif1α* ESTs map to LG4 in a region predicted to be syntenic to the region of human Chromosome 7 that contains the *TIF1α* gene based on the conserved locations of eight other orthologous gene pairs, including *SEMA3A,* mapped to these regions in human and zebrafish (Barbazuk et al. 2000). We next compared the embryonic expression pattern of *tif1α* mRNA to *tif1γ* by in situ hybridization. Like mammalian *TIF1α* (Le Douarin et al. 1995; Niederreither et al. 1999), the predicted zebrafish *tif1γ* gene is broadly expressed (see Figure 4A). At five somites, zebrafish *tif1α* does not display the relatively high expression in the horseshoe-shaped region of hematopoietic mesoderm seen with *tif1γ.* At later stages, *tif1α* is evenly expressed throughout most of the embryo, including the developing blood islands. Therefore, *tif1α* is coexpressed in the same cells with *tif1γ* and may therefore be available to form hetero-oligomers in vivo.

### Forced Expression of *tif1γ* Rescues Hematopoiesis in *mon* Mutants

**Figure 5:**
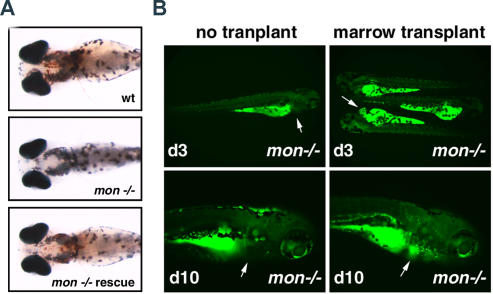
Overexpression of Wild-Type *tif1γ* mRNA or Marrow Transplantation Rescues Embryonic Hematopoiesis in *mon* Mutants (A) *mon^tg234^* mutants are rescued by injection of mRNA-encoding wild-type Tif1γ protein. At 4 d of development, large numbers of RBCs are visible in the circulation of wild-type zebrafish, shown here by o-dianisidine staining of hemoglobin. Uninjected *mon^ttg234^* homozygous mutants are completely bloodless. Injection of 100 pg of wild-type *tif1γ* mRNA rescues erythropoiesis in mutant embryos. o-dianisidine-stained larvae are shown in ventral views to highlight blood in vessels. (B) Transplantation of wild-type zebrafish marrow cells carrying a *gata1:GFP* transgene into 2-d-old embryos reconstitutes erythropoiesis, but not viability, in *mon^tg234^* homozygous mutants. Still frames from movies of live embryos at day 3 posttransplant highlight less than 100 GFP^+^ RBCs in circulation (top). Transplanted cells were observed to proliferate resulting in thousands of donor-derived erythrocytes 7 d later (bottom). Arrows indicate the hearts of control and transplanted zebrafish. See Videos S1–S4.

To further confirm that a mutation in the zebrafish *tif1γ* gene is responsible for the *mon* mutant phenotype we performed embryo rescue experiments (Figure 5A; Table 1). Microinjection of synthetic wild-type *mon* mRNA at the one-cell stage rescues the formation of embryonic erythrocytes in genotyped mutant embryos without causing obvious defects in embryonic patterning or organogenesis. At 4 d of development, 70% (*n* = 10) of *mon^tg234^* mutants show significant (greater than 200 cells in comparison to a wild-type estimate of 3,000 cells) rescue of circulating hemoglobinized RBCs in comparison to control sibling mutants (*n* = 75). Based on the correction of the jagged fin-fold phenotype (Ransom et al. 1996), the mesenchymal cells are rescued to a similar extent as the anemia (unpublished data). Overexpression of *mon* did not result in expanded blood cell numbers in wild-type embryos and was not toxic at doses that rescue the phenotype of *mon* mutants (unpublished data). Since there were no expanded or ectopic blood populations in the embryos, these rescue experiments suggest that *mon* functions as a permissive factor required for hematopoiesis.

**Table 1:**
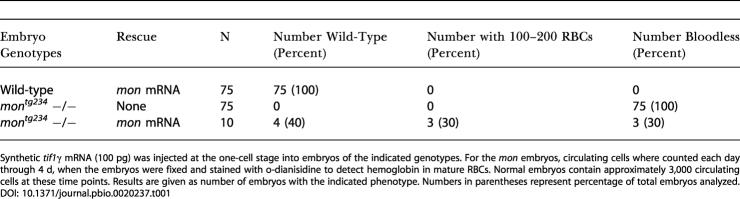
Overexpression of *tif1γ* mRNA Rescues *mon* Mutants: Hematopoietic Phenotypes

Synthetic *tif1γ* mRNA (100 pg) was injected at the one-cell stage into embryos of the indicated genotypes. For the *mon* embryos, circulating cells where counted each day through 4 d, when the embryos were fixed and stained with o-dianisidine to detect hemoglobin in mature RBCs. Normal embryos contain approximately 3,000 circulating cells at these time points. Results are given as number of embryos with the indicated phenotype. Numbers in parentheses represent percentage of total embryos analyzed

### Marrow Transplantation Rescues Erythropoiesis in *mon* Mutants

The high levels of *tif1*γ expression in erythroid cells suggest that it functions as a cell-autonomous regulator of gene expression in hematopoietic cells. In order to test this hypothesis, we transplanted wild-type adult zebrafish kidney marrow cells carrying a *gata1:green fluorescent protein (GFP)* transgene into 48-hpf *mon* mutant embryos (Figure 5B; Table 2). The *gata1:GFP* transgene makes use of the *gata1* promoter to drive GFP expression and can thus be used to mark donor-derived erythroid cells (Long et al. 1997). Untransplanted mutant embryos have no embryonic blood cells in circulation. Following transplantation, mutant host embryos were observed daily for 2 wk. Of 191 mutant embryos injected, 129 (68%) showed GFP^+^ cells in circulation 2 d later. Many recipients showed robust increases in donor-derived cells over the observation period. Of 81 recipients initially scored as having less than ten GFP^+^ cells at day 2 posttransplant, 13 (16%) of these demonstrated a marked increase in erythroid cells with 100–1,000 GFP^+^ cells in circulation 6 d later. By day 10, these transplanted embryos showed approximately 3,000 cells in circulation, similar to the number of blood cells in normal embryos. Despite robust reconstitution of blood cells, mutant recipients did not inflate their swim bladders and thus failed to survive longer than nontransplanted sibling controls, all dying by 3 wk of age. In contrast, 13/35 (37%) heterozygous *mon^tg234^* transplants survived to early adulthood. Similar transplants of wild-type cells can fully rescue *vlad tepes (gata1)* mutants (Traver et al. 2003). Therefore, the results of cell transplantations suggests that *tif1γ* plays a cell-autonomous role in erythroid cells, and its role in nonhematopoietic tissues, such as trunk mesenchyme or the nervous system, is also required for embryo survival.

**Table 2:**
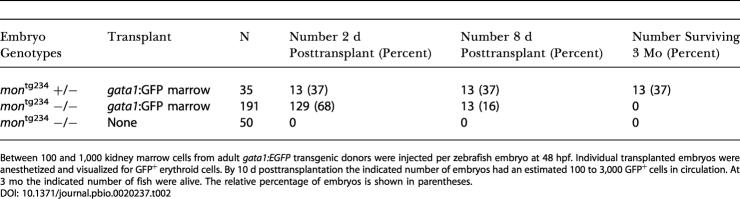
Marrow Transplantation Rescues Hematopoiesis But Not Survival in *mon* Mutants: Embryos with Transplanted Erythroid Cells

Between 100 and 1,000 kidney marrow cells from adult *gata1:EGFP* transgenic donors were injected per zebrafish embryo at 48 hpf. Individual transplanted embryos were anesthetized and visualized for GFP^+^ erythroid cells. By 10 d posttransplantation the indicated number of embryos had an estimated 100 to 3,000 GFP^+^ cells in circulation. At 3 mo the indicated number of fish were alive. The relative percentage of embryos is shown in parentheses

### Tif1γ in Punctate Nuclear Foci Is Developmentally Regulated

**Figure 6:**
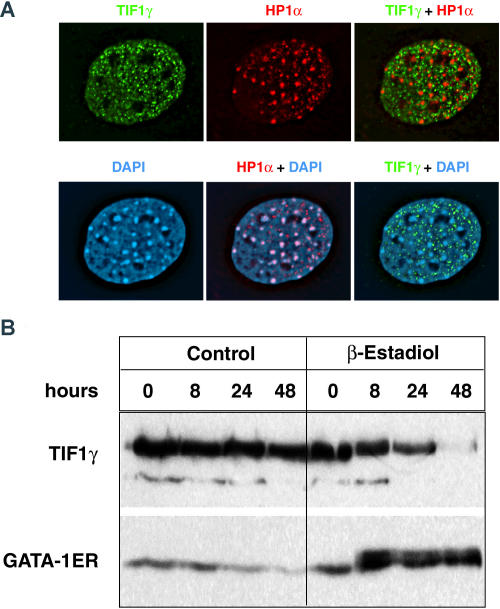
Mammalian Tif1γ Protein Localizes to Nuclear Bodies Distinct from Heterochromatin (A) Deconvolved immunofluorescence images of a mouse embryonic fibroblast cell nucleus stained with an anti-Tif1γ antibody and stained with a monoclonal antibody directed against HP1α. This is also compared to DAPI staining. The merged images of the nucleus show that Tif1γ does not colocalize with the HP1α or DAPI staining of heterochromatin while HP1α and DAPI staining overlap. (B) G1ER mouse erythroleukemia cells express high levels of Tif1γ protein as detected by Western blot analysis. Expression of Tif1γ decreases during Gata1-dependent erythroid maturation induced by β-estradiol treatment to induce a Gata1–ER fusion protein.

In order to examine the subcellular distribution of Tif1γ protein, we generated an affinity-purified rabbit polyclonal antiserum directed against the C-terminal 15 amino acids conserved in human TIF1γ and mouse Tif1γ. Immunofluorescence of mouse embryo fibroblast nuclei with the anti-Tif1γ antiserum demonstrates that Tif1γ is localized in small nuclear foci (Figure 6A). The localization of Tif1γ protein appears different from the more diffuse nuclear patterns typically seen in studies of Tif1α (Remboutsika et al. 2002) or TIF1β (Cammas et al. 2002). A recent report demonstrates that TIF1β associates with heterochromatin-containing foci after retinoic acid treatment or serum starvation (Cammas et al. 2002). Thus, localization or expression of the TIF1 proteins may be regulated during distinct developmental processes or by environmental cues. The nuclear foci that contain Tif1γ do not colocalize with two markers of heterochromatin, HP1α protein and DAPI staining of DNA (Figure 6A). Furthermore, Tif1γ does not colocalize with promyelocytic leukemia gene product (PML) nuclear bodies, DNA repair complexes that contain Mre11, or transcriptional complexes containing TFII-B (unpublished data). We next examined the expression of Tif1γ protein during the differentiation of G1E cells, a murine erythroleukemia cell line that can terminally differentiate into erythrocytes when a Gata1:estrogen receptor fusion protein is stabilized in response to estrogen exposure (Weiss et al. 1997). Western blot analysis demonstrated that Tif1γ protein expression decreases with terminal erythroid differentiation (Figure 6B). Consistent with this finding, after 24 hpf, zebrafish *mon* mRNA expression falls during the terminal maturation of the primitive erythroid cells (unpublished data). In two different murine erythroleukemia cell lines (MEL and G1E), Tif1γ is also expressed in nuclear foci, and even though the overall Tif1γ protein level is reduced, this nuclear foci localization does not change with differentiation (unpublished data). This provides further support for the hypothesis that Tif1γ acts within novel nuclear foci, during erythroid differentiation.

## Discussion

The zebrafish is an excellent model system to elucidate the molecular machinery controlling gene expression during hematopoiesis (Thisse and Zon 2002; Galloway and Zon 2003). As part of a large-scale forward genetic screen, we originally identified a complementation group of independent mutant alleles in the zebrafish gene that we named *moonshine* (Ransom et al. 1996). Positional cloning was used to identify the *mon* gene, establishing a critical role for a transcriptional intermediary factor, Tif1γ, during hematopoietic development.

### The *mon* Gene Encodes the Zebrafish Ortholog of Mammalian TIF1γ

Our results strongly support the conclusion that we have positionally cloned the zebrafish *mon* gene correctly, and it is the ortholog of mammalian Tif1γ. Tif1γ is present in the critical genetic interval encompassing a single approximately 50-kb PAC clone defined by linkage analysis (see Figure 3). Sequence analysis indicates that zebrafish *tif1γ* is most similar in predicted amino acid sequence and intron/exon structure compared to the predicted orthologous human and mouse genes. Zebrafish *tif1γ* is located in a region of zebrafish Chromosome 8 syntenic to the region of human Chromosome 1 containing *TIF1γ.* We identified point mutations in *tif1γ* from three different alleles of *mon* that each result in premature stop codons and mRNA decay. In addition, *tif1γ/Tif1γ* is highly expressed in hematopoietic cells throughout embryogenesis in both zebrafish and mouse (see Figure 4). And as predicted, forced expression of wild-type *tif1γ* mRNA efficiently rescues hematopoiesis in *mon* mutants and does not perturb hematopoiesis in wild-type embryos (see Figure 5). We have also cloned the predicted zebrafish ortholog of *tif1α,* which is more uniformly expressed in zebrafish embryos like mammalian *TIF1α* (Le Douarin et al. 1995; Niederreither et al. 1999) (see Figures 3A and 4A) and may therefore be available to form hetero-oligomers with Tif1γ protein in developing hematopoietic cells. Comparing available zebrafish and mammalian TIF1-predicted amino acid sequences, it appears that the Tif1γ orthologs are the most highly conserved family members while the Tif1α sequences are relatively more divergent. We have not found a Tif1β ortholog, thus far, in the zebrafish or *fugu* genome or EST sequences. It is possible that Tif1β, like the KRAB domain transcription factors it binds to, may be present only in tetrapods (Urrutia 2003). However, more complete genome sequences will be needed to confirm this hypothesis. Based on our analysis of zebrafish *mon* mutants, it is reasonable to predict that Tif1γ, the most evolutionarily conserved TIF1 family member, plays a similarly essential role in human and mouse hematopoiesis.

### Mutations in *tif1γ* Cause Apoptosis of Erythroid Progenitors

Our examination of hematopoietic gene expression, apoptosis, and marrow histology in *mon* mutants demonstrates that early erythroid progenitors are formed in homozygous mutants, but they fail to properly differentiate and instead undergo programmed cell death (see Figure 1). The expression of *gata1* appears to initiate normally in the committed erythroid cells of *mon* mutants. However, the cells are abnormal prior to the complete loss of *gata1* expression. TUNEL-positive apoptotic cells are abundant by the 12-somite stage of development, and by 22 hpf all hematopoietic gene expression is extinguished. The expression of marker genes, including *scl* and *gata2,* characteristic of hematopoietic stem cells and primitive hematopoietic progenitors, are also not detected in the embryonic blood islands of mutants at 22 hpf. This indicates that the mutant hematopoietic cells are not blocked prior to commitment to the erythroid lineage, but instead develop as abnormal erythroid cells and undergo apoptosis, similar to *gata1-*deficient erythroid cells (Fujiwara et al. 1996; Lyons et al. 2002). Defective erythropoiesis and severe anemia were also observed in rare surviving homozygous mutant *mon* adults, demonstrating that *tif1γ* is also required in definitive hematopoiesis (see Figure 2).

The zygotic phenotypes of *mon* mutants may not reveal the function of maternally inherited Tif1γ. Maternally expressed zebrafish Tif1γ may play roles in hematopoiesis or other aspects of organogenesis that are not detectable due to the presence of wild-type mRNA in eggs laid by heterozygous mothers. Analysis of the offspring of homozygous *mon* mutant female zebrafish will aid in defining the function of this maternal mRNA. The present analysis of zygotic *mon* mutants provides data that are consistent with the conclusion that *tif1γ* is essential for erythropoiesis but do not rule out essential functions in other hematopoietic lineages.

The hematopoietic phenotype of *mon* mutants resembles the loss of erythroid cells seen in both mouse *Gata1* knockout embryos and zebrafish *vlad tepes (gata1)* mutant embryos (Fujiwara et al. 1996; Lyons et al. 2002). In an effort to determine if there is a genetic relationship between *mon* and *gata1,* we tested their ability to rescue erythropoiesis. Both injection of *gata1* mRNA into *mon* homozygous mutant embryos and injection of *tif1γ* mRNA into *gata1* knock-down embryos failed to rescue hematopoiesis (unpublished data). We also tested for a direct interaction between Tif1γ and Gata1 proteins by coimmunoprecipitation and yeast two-hybrid assays and found no association (unpublished data). Although the mutations in each of these genes arrest cells at a similar stage of development, our results suggest that *gata1* and *tif1γ* act independently. This does not rule out the possibility that parallel genetic pathways involving *gata1* and *tif1γ,* operating together, regulate gene transcription within blood cells.

### The Role of Tif1γ in Primitive and Definitive Erythropoiesis

Taken together, our data suggest that *tif1γ* is required as a permissive cofactor for the erythroid lineage-specific control of hematopoietic gene expression. We reasonably predict that Tif1γ protein functions as a transcriptional intermediary factor in hematopoietic progenitor cells given that both TIF1α (Zhong et al. 1999) and TIF1β (Friedman et al. 1996; Abrink et al. 2001) have been shown to act as intermediary factors that positively or negatively regulate gene transcription. These studies indicate that TIF1α and TIF1β act as scaffolds that link different classes of DNA-binding proteins and chromatin-associated proteins into larger regulatory complexes. Tif1γ is detected within nuclear foci (see Figure 6), which, based on our analysis, do not appear to correspond to several types of previously described nuclear bodies, including PML bodies. Localization of Tif1γ to these nuclear bodies may be regulated by posttranslational modification such as SUMO modification that is required for PML to form PML nuclear domains (Zhong et al. 2000a, 2000b; Best et al. 2002). These foci may serve as assembly points where Tif1γ forms multisubunit complexes with DNA-binding transcription factors and their other essential coactivators or corepressors, during the early stages of erythroid differentiation. It will be important to determine the identity of Tif1γ-interacting proteins in nuclear foci and establish how they function with Tif1γ to regulate blood cell development.

## Materials and Methods

### 

#### Zebrafish and mouse strains and studies

Zebrafish were maintained and staged as described (Westerfield 1998). The alleles *mon^tb222b^* and *mon^tg234^* were generated in a large-scale screen for ENU-induced mutations (Ransom et al. 1996) on the TU, whereas the *mon^m262^* allele was derived on the AB strain and was originally called *vampire* (Weinstein et al. 1996). Mapping strains were constructed by mating to WIK or SJD polymorphic strains. Linkage analysis was performed on haploid or diploid embryos obtained from TU/SJD or TU/WIK hybrids. In situ hybridization and stainings of embryos were done as described (Thompson et al. 1998; Liao et al. 2002). In situ hybridization of mouse embryos was performed as described (Kingsley et al. 2001). Genomic DNA isolation, genotyping, AFLP analysis, and chromosomal walking were each performed as previously described (Brownlie et al. 1998; Ransom and Zon 1999). A complete list of primers for genetic mapping, RT-PCR, and sequencing of *mon* are available on request.

#### mRNA expression constructs, morpholinos, and microinjection

The full-length *mon* cDNA was subcloned into EcoRI and XhoI sites in the pCS2^+^ vector. Synthetic mRNA was transcribed in vitro, and microinjection was performed essentially as described (Liao et al. 2002).

#### Cell transplantation

Whole kidney marrow cells were isolated from adult *gata1:EGFP* transgenic donors, resuspended in 0.9X phosphate-buffered saline + 5% fetal bovine serum, and injected into the sinus venosus of 2-d-old mon^tg234^ −/− and control embryos. Between 10^2^ and 10^3^ kidney marrow cells were injected per embryo. Individual transplanted embryos were anesthetized and visualized daily under an inverted fluorescent microscope (DM-IRE2; Leica, Wetzlar, Germany) for GFP^+^ cells over a span of 12 d. On day 13 posttransplant, all surviving larvae (12/129; 9%) were placed in tanks and monitored for survival.

#### Antibodies, immunostaining, and immunoblots

Antisera against the human C-terminal TIF1γ sequence RRKRLKSDERPVHIK was generated in rabbits (Genemed Synthesis, South San Francisco, California, United States) and affinity purified. Mouse embryonic fibroblasts grown on coverslips were immunostained with HP1α (Chemicon, Temecula, California, United States) and Tif1γ antisera simultaneously. In brief, cells were fixed in 4% paraformaldehyde for 5 min, washed with phosphate-buffered saline, and blocked with 5% serum (PBST) for 30 min. After incubation with the primary antibodies (PBST, 60 min) cells were washed three times with PBST and incubated with secondary antibodies (Jackson Laboratory, Bar Harbor, Maine, United States) followed by three washes in PBST. Cells were embedded with Vectashield/DAPI and analyzed using an Axioplan 2 microscope (Zeiss, Jena, Germany). Digital images were processed using the Volocity 1.0 software (Improvision, Lexington, Massachusetts, United States). G1E cell differentiation experiments were performed essentially as described (Weiss et al. 1997).

## Supporting Information

Transplantation of wild-type zebrafish marrow cells carrying a *gata1:GFP* transgene into 2-d-old embryos reconstitutes erythropoiesis, but not viability, in mon^tg234^ homozygous mutants. Movies of live embryos at day 3 posttransplant highlight less than 100 GFP^+^ RBCs in circulation. Transplanted cells were observed to proliferate, resulting in thousands of donor-derived erythrocytes 7 d later. Movies present GFP-fluorescent images of live zebrafish larvae.

Video S1 Untransplanted Control *mon^tg234^* Homozygous Mutants Had No Fluorescent Cells in Circulation at 3 Days of Development (13.7 MB MOV).

Video S2 One Day after Transplantation, Less Than 100 GFP^+^ Erythrocytes Were Visible in the Circulation of Three *mon^tg234^* Homozygous Mutants (11.3 MB MOV).

Video S3 Untransplanted Control *mon^tg234^* Homozygous Mutants Had No Fluorescent Cells in Circulation at 9 Days of Development (7.9 MB MOV).

Video S4 Seven Days after Transplantation, Thousands of Donor-Derived Erythrocytes Were Visible in the Circulation of a Representative *mon^tg234^* Homozygous Mutant (11.2 MB MOV)

### Accession Numbers

The GenBank (http://www.ncbi.nlm.nih.gov/Genbank) accession numbers for the genes and gene products discussed in this paper are fly bonus (AAF19646), human TIF1α (015164), human TIF1β (Q13263), human TIFγ (Q9UPN9), human TIF1γ (Q9UPN9), *mon* (AY59853), mouse Tif1α (Q64127), mouse Tif1β (AAH58391), and mouse Tif1γ (NP444400).

The cDNA sequences of zebrafish *mon/tif1γ* and *tif1α* have been deposited in GenBank under the accession numbers AY598453 and AY598454, respectively.

## Abbreviations

AFLP: amplified fragment length polymorphism
ENU: ethyl-nitrosourea
EST: expressed sequence tag
GFP: green fluorescent protein
hpf: hours postfertilization
KRAB: Krüppel-associated box
*mon*: *moonshine;* PAC
PML: promyelocytic leukemia gene product
RBCs: red blood cells
*scl*: *stem cell leukemia;* SSCP
TIF: transcriptional intermediary factor
TU: Tübingen background
